# Resolving the links between efferocytosis and *DNMT3A* mutation–driven clonal hematopoiesis in atherosclerosis

**DOI:** 10.1172/JCI211265

**Published:** 2026-10-15

**Authors:** Aarti Asnani

**Affiliations:** Beth Israel Deaconess Medical Center and Harvard Medical School, Boston, Massachusetts, USA.

## Abstract

Efferocytosis, or the clearance of apoptotic cells by macrophages, has been implicated in tissue resolution that promotes disease regression in atherosclerosis and other diseases of chronic inflammation. The mechanisms by which efferocytosis leads to pro-resolving macrophage reprogramming have not been fully defined. In this issue of the *JCI*, Avrampou and colleagues found that efferocytosis activates DNA-methyltransferase-3A (DNMT3A) to promote base excision repair and suppress oxidized DNA. Moreover, they provide evidence that DNMT3A enabled tissue repair in the context of plaque regression in a mouse model of atherosclerosis. Human studies have linked *DNMT3A* mutation–mediated clonal hematopoiesis (DNMT3A-CH) with atherosclerosis. The findings of Avrampou et al. linking DNMT3A function to efferocytosis-mediated tissue repair suggest that this pathway could be modulated therapeutically to minimize cardiovascular events in patients.

## Pro-resolving mediators in atherosclerosis

Atherosclerosis and its sequelae represent the leading cause of mortality worldwide. Given the central role of chronic inflammation in the development of atherosclerotic plaque, preclinical and clinical studies have largely focused on the use of antiinflammatory therapies, such as statins, colchicine, and IL-6–directed treatments to mitigate downstream cardiovascular events. In parallel, pro-resolving therapies may be equally important to mitigate inflammation and promote tissue repair (1). Several classes of endogenous specialized pro-resolving mediators have been implicated in vascular disease, including resolvins, protectins, and maresins, all of which function to reprogram immune cell subsets toward the resolution of inflammation (2). In addition to their roles in limiting neutrophil recruitment and counterregulating inflammatory cytokines, these pro-resolving mediators enhance macrophage-mediated clearance of apoptotic cells, a process known as efferocytosis (3).

Prior work has shown that specialized pro-resolving mediator levels are decreased in vulnerable atherosclerotic plaques and that treatment with resolvins can improve efferocytosis and promote plaque stability in mouse models of atherosclerosis (4). Macrophages take up amino acids, including methionine, during efferocytosis (5). Apoptotic cell–derived methionine is converted to S-adneosylmethionine, which then activates DNTM3A-mediated DNA methylation, promoting ERK1/2–TGF-β1 signaling in efferocytosing macrophages to enhance tissue resolution (6). Notably, *DNMT3A* is the most frequently mutated gene in clonal hematopoiesis (CH), which leads to the expansion of leukocytes with acquired somatic mutations. *DNMT3A* mutation–driven CH (DNMT3A-CH) has been linked to a higher risk of coronary heart disease in humans and accelerated atherosclerosis in mouse models (7, 8) through what appears to be an IL-6–independent mechanism, based on human genetic data (9). A prior report implicated DNMT3A’s role in cGAS-type I interferon signaling in macrophages isolated from human atherosclerotic plaques (10), although the exact mechanisms by which DNTM3A-CH may contribute to atherosclerosis have not been fully established.

## Efferocytosis activates DNTM3A-mediated base excision repair in atherosclerosis

In this issue of the *JCI*, Avrampou and colleagues build upon these prior observations to establish a role for DNMT3A-mediated DNA methylation in efferocytosis in atherosclerotic plaques (11). In mouse and human macrophages, they unexpectedly observed that efferocytosis activated base excision repair to remove 8-hydroxy-2’-deoxyguanosine (8-OHdG) from DNA in a DNMT3A-dependent manner, thereby promoting efferocytosis-induced macrophage proliferation. Efferocytosis also enhanced chromatin binding of poly(ADP-ribose) polymerase 1 (PARP1) to facilitate formation of the base excision repair complex and increased nuclear MutT homolog 1 (MTH1) to hydrolyze excise 8-OHdG. Both of these processes required DNMT3A. Using dexamethasone-induced thymocyte apoptosis to model efferocytosis-induced tissue resolution, they demonstrated that the DNMT3A-dependent efferocytosis pathway was integral to thymic repair.

Finally, the authors set out to determine how this DNMT3A-dependent mechanism affects atherosclerosis progression. They developed an *Ldlr^–/–^* mouse model of DNMT3A-CH to highlight the relevance of this pathway in atherosclerotic regression–induced cap thickening, an endpoint of LDL-lowering treatments that reflects plaque stabilization. Use of an LDL-lowering regimen led to marked thickening of the fibrous cap and elevated expression of pro-resolving plaque macrophage markers in atherosclerotic WT mice, whereas atherosclerotic DNMT3A-CH mice demonstrated markedly less cap thickening and low-to-no increase in marker expression (Figure 1). Taken together, these findings implicate DNA repair as a mechanism linking apoptotic cell–derived methionine to DNMT3A-mediated DNA methylation and pro-resolving macrophage proliferation in the context of atherosclerosis.

## Future directions and opportunities for translation

Beyond atherosclerosis, DNMT3A mutation in human heart failure has been associated with upregulation of inflammatory pathways in monocytes that led to changes in cytokine and T cell profiles (12). Additionally, DNTM3A has been shown to play a role in cardiac fibrosis via paracrine effects on local fibroblasts (13). Moving forward, it will be informative to define the extent to which DNMT3A-mediated DNA methylation and the resulting effects on efferocytosis observed in atherosclerotic plaques may overlap or synergize with the inflammatory pathways and cell-cell crosstalk observed in heart failure. Comprehensive single-cell profiling could provide an opportunity for further characterization of the discrete macrophage populations involved in DNMT3A-CH–associated atherosclerosis, lending insight into how best to modulate these pathways therapeutically.

The findings from Avrampou et al. (11) build on the growing recognition that CH contributes to systemic inflammation in cardiovascular disease. As highlighted by the authors, the specific *DNTM3A* mutation studied in this work (R882H in humans) confers loss of function in the methyltransferase domain and may therefore have higher specificity for cardiovascular disease phenotypes in patients relative to other *DNTM3A* variants associated with CH. Notably, Avrampou et al. (11) propose a link between DNMT3A-CH and the tissue resolution pathways that play a role in atherosclerosis regression induced by statins (see discussion in ref. 14), highlighting the potential for these findings to direct the use of lipid-lowering therapies at the population level. Further investigation of the effects of lipid-lowering interventions in humans with DNMT3A-CH, particularly in those carrying the R882H variant, could enable translation of the mechanisms driving efferocytosis-mediated tissue repair to the clinic.

## Conflict of interest

AA is a cofounder and member of the board of directors of Corventum Inc. She receives royalties from patent US20210163495A1 (Tricyclic compounds as Cyp1 Inhibitors).

## Figures and Tables

**Figure 1:**
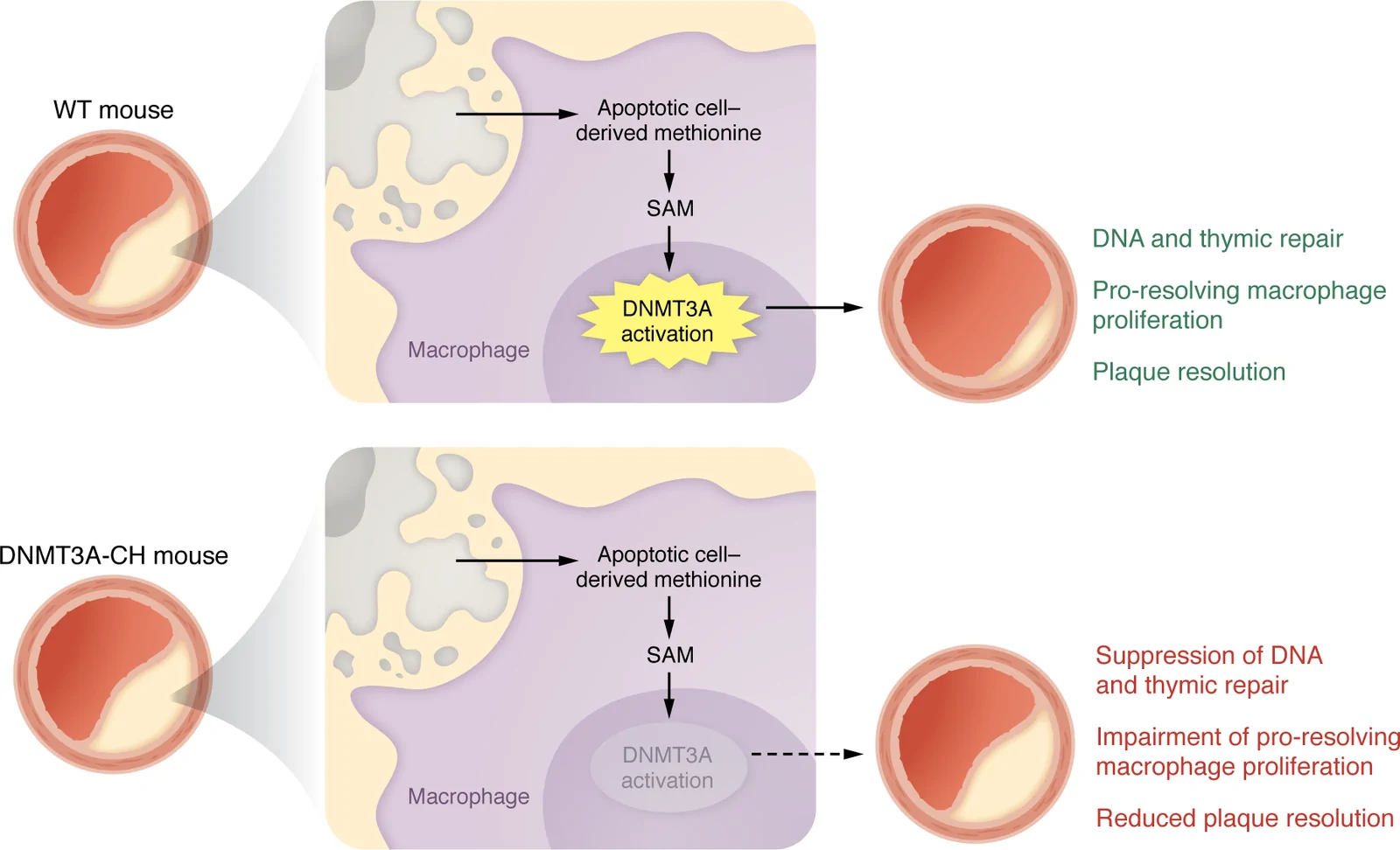
DNMT3A activation is linked to DNA repair, efferocytosis, and plaque resolution in a mouse model of atherosclerosis. Avrampou et al. (11) observed that efferocytosis stimulated proliferation of pro-resolving macrophages through a base excision repair pathway that requires DNMT3A activation. Noting that *DNMT3A* mutation–driven clonal hematopoiesis (DNMT3A-CH) is associated with coronary heart disease and atherosclerosis (7, 8), the authors found that LDL-lowering regimens failed to induce atherosclerotic plaque stabilization in DNMT3A-CH mice. These findings solidify the connection between DNMTA3 function, efferocytosis, and resolution within plaques. SAM, S-adenosylmethionine.

## Version 1 10/15/2026

Electronic publication

## References

[1] Serhan CN, Savill J (2005). Resolution of inflammation: the beginning programs the end. Nat Immunol.

[2] Conte MS (2018). Pro-resolving lipid mediators in vascular disease. J Clin Invest.

[3] Doran AC (2020). Efferocytosis in health and disease. Nat Rev Immunol.

[4] Fredman G (2016). An imbalance between specialized pro-resolving lipid mediators and pro-inflammatory leukotrienes promotes instability of atherosclerotic plaques. Nat Commun.

[5] Yurdagul A (2020). Macrophage metabolism of apoptotic cell-derived arginine promotes continual efferocytosis and resolution of injury. Cell Metab.

[6] Ampomah PB (2022). Macrophages use apoptotic cell-derived methionine and DNMT3A during efferocytosis to promote tissue resolution. Nat Metab.

[7] Jaiswal S (2017). Clonal hematopoiesis and risk of atherosclerotic cardiovascular disease. N Engl J Med.

[8] Rauch PJ (2023). Loss-of-function mutations in Dnmt3a and Tet2 lead to accelerated atherosclerosis and concordant macrophage phenotypes. Nat Cardiovasc Res.

[9] Vlasschaert C (2023). Interleukin-6 receptor polymorphism attenuates clonal hematopoiesis-mediated coronary artery disease risk among 451 180 individuals in the UK Biobank. Circulation.

[10] Cobo I (2022). DNA methyltransferase 3 alpha and TET methylcytosine dioxygenase 2 restrain mitochondrial DNA-mediated interferon signaling in macrophages. Immunity.

[11] Avrampou K (2026). Efferocytosis activates a DNMT3A-mediated oxidized DNA repair pathway to enable tissue resolution. J Clin Invest.

[12] Abplanalp WT (2021). Clonal hematopoiesis-driver DNMT3A mutations alter immune cells in heart failure. Circ Res.

[13] Shumliakivska M (2024). DNMT3A clonal hematopoiesis-driver mutations induce cardiac fibrosis by paracrine activation of fibroblasts. Nat Commun.

[14] Moreno PR (2014). Strengthening the Achilles heel of high-risk plaques. J Am Coll Cardiol.

